# Designing and Psychometric Evaluation of Iranian Students’ Academic Stress Questionnaire (IAASQ)

**Published:** 2020-04

**Authors:** Zahra HOSSEINKHANI, Saharnaz NEDJAT, Mahboubeh PARSAEIAN, Fatemeh VEISI HAMPA, Hamid-Reza HASSANABADI

**Affiliations:** 1. Department of Epidemiology and Biostatistics, School of Public Health, Tehran University of Medical Sciences, Tehran, Iran; 2. Department of Nutrition, School of Health, Qazvin University of Medical Sciences, Qazvin, Iran; 3. Department of Educational Psychology, Kharazmi University, Tehran, Iran

**Keywords:** Stress, Adolescents, Questionnaire, Factor analysis, Iran

## Abstract

**Background:**

Academic stress is one of the factors affecting the health of adolescents. The aim of present study was to design an academic stress questionnaire for Iranian adolescents with regard to the cultural and educational system of the country.

**Methods:**

After reviewing the literature and identifying available tools in 2018, Iranian adolescents’ views on academic stress were extracted. Similar questions have been used in other tools. According to expert opinion, the results were overviewed and the initial version was designed. The steps of content validity and instrument reliability were carried out. Internal consistency was investigated with Cronbach’s alpha (α) and repeatability with Intra Class Correlation (ICC). After dividing the data into two randomized samples, exploratory factor analysis (EFA) with 899 subjects and confirmatory factor analysis (CFA) with 717 subjects were performed. The association between this tool and the Morgan and Jink’s Self Efficacy Scale and the Strengths and Difficulties Questionnaire questionnaires was investigated. Data were analyzed using SPSS and Mplus softwares.

**Results:**

The Iranian Adolescent Academic Stress Questionnaire (IAASQ) was designed with 57 questions. Relevancy and clarity of the whole tool were obtained as 0.81 and 0.83, respectively. In different domains, Cronbach’s alpha was in the range (0.58–0.85) and ICC (0.80 (95% CI:0.66–0.90)). In the EFA, 9 factors were extracted. CFA confirmed the suitability of the model in another sample. Discriminant and convergent validity tool was approved.

**Conclusion:**

The IAASQ questionnaire has acceptable reliability and validity. This tool is recommended for use in related studies in the Iranian community.

## Introduction

Academic stress is a particular type of stress appeared due to the expectations of parents and teachers from students because of academic achievement (1). Students who are happy in their lives and have less stress will do their home works better and achieve more success, while those with less physical and mental health have less motivation to study and use methods such as killing the time and absence from school to get along with academic stress. Generally, daily stresses, mostly influenced by the amount of social support of individuals and is a risk factor for adolescents’ mental health, have a positive relationship with academic failure and reduce the happiness of individuals (2–4).

Several studies have examined the academic stress of adolescents in different countries and mentioned various factors such as inability to fulfill parents’ expectations of education, pressure from peers, wrong comparison with peers, and concerns about future decisions in life as factors of academic stress (5–17).

In Iran, in addition to the above mentioned, the anxiety about the failure to pass the university entrance exam (Konkour) is considered as one of the most important causes of academic stress, which encompasses a wide range of educational grades (8,15).

Since academic stress is affected by many issues such as cultural-social conditions, family circumstances and educational system in any country, in order to reduce academic stress among adolescents, in addition to measuring it in different domains, the role of other stressors in adolescence also should be noted that in different studies, this issue and the role of each of the different domains of academic stress have been less considered. Of course, Byrne et al. have developed the Adolescents Stress Questionnaire (ASQ) that examines stresses during adolescence with 58 questions in 10 different domains (9,18–20) and it has also been used in the present study. Therefore, the aim of this study was to design an Iranian adolescent academic stress questionnaire with regard to various influencing domains including cultural-social factors and educational system of the country.

## Methods

This cross-sectional study was conducted with the aim of designing an Iranian adolescent academic stress questionnaire.

### Development of questionnaire

First reviewing the literature was done to prepare the Iranian adolescent academic stress questionnaire. Questionnaires existing in this field were identified (9,21–25). Then, regarding the educational system of the country and the existing cultural-social aspects, the viewpoint of adolescents about academic stress in Iranian society was investigated in a qualitative study of directional content analysis. This study was conducted by holding semi-structured interviews with 43 adolescents. Participants from both genders aged 12 to 18 yr old were from different schools (public, private, and special schools) and all economic groups. Since the most of the adolescent academic stress questionnaires effective factors had been studied in general and with a limited number of questions, the Adolescents Stress Questionnaire (ASQ), which was similar to the objectives of the present study, was considered. This questionnaire measures the adolescent stress in 10 different domains of academic and adolescence stress, including family life stress, school homework, attendance at school, romantic relationships, pressure from peers, interaction with teacher, future ambiguity, school/amusement contradiction, financial pressure, and adolescent responsibility (9,18,20,26,27). Therefore, in order to provide Iranian tool, while summarizing the views of participants in a qualitative study confirmed by expert opinion, questions of different domains of academic stress of Iranian society that was in common with the ASQ questionnaire were translated by backward-forward method and the different dimensions of academic stress in Iranian society and the ASQ questionnaire was adapted.

### Content Validity

After preparing the initial version for determining the content validity, the questionnaire was given to 10 content experts including educational psychologist and epidemiologist and lay expert including students, parents and teachers. They were asked to give a qualitative comment on the questions and score relevancy and clarity of the questions, from 1 to 4 (1-undesirable, 2-partially favorable, 3-desirable and 4-totally undesirable). Relevancy and Clarity Indicators of each item was calculated using the Item Content Validity Index (I-CVI) and the entire tool with the Scale Content Validity Index (S-CVI/Average) approach (28).

### Reliability

Twenty-five students completed the questionnaire twice within 3 wk interval. Internal consistency and repeatability of the findings were calculated with Cronbach’s alpha (α) and Intra Class Correlation (ICC), respectively.

### Construct Validity

This study was conducted in the academic year 2017–18 with stratified multi-stage sampling in high school students in city of Qazvin. First, each school was considered as a strata, then it was randomly selected as the systematic method. The number of students’ required (30 subjects) was randomly selected from all educational backgrounds, and the data was collected. The data were then randomly divided into two parts. The first sample was studied for exploratory factor analysis (899 out of 30 schools) and the second sample was studied for confirmatory factor analysis (717 out of 24 schools).

### Exploratory Factor Analysis

To do the exploratory factor analysis, sampling was measured with the Kaiser-Meyer-Olkin’s (KMO) which compare the zero-order correlation matrix of the variables with the partial correlation matrix of the variables. The correlation matrix was tested by Bartlett’s method, which tests the hypothesis of zero on the homogeneity of the correlation matrix of society. Questions that were cross-loading were deleted. The loading factor was considered as 0.4 for exploration of the factors (29,30). In the analysis, EFA extraction was performed using the principal components method and rotation with the varimax method. The analysis was performed using SPSS software, version 18(Chicago, IL, USA). The significance level was considered as 0.05.

### Confirmatory factor Analysis

In order to verify the factor structure of the questionnaire, the confirmatory factor analysis was performed in another sample of Iranian adolescents. The maximum likelihood method was used to estimate the parameters. The indicators used to measure the goodness of fit were χ^2^ and root mean square error approximation (RMSEA). Data were analyzed with Mplus 7 software.

### Discriminant and Convergent Validity

To examine the convergent validity and discriminant questionnaire, MJSES questionnaire (Morgan and Jink’s Self Efficacy Scale) and SDQ questionnaire (the Strengths and Difficulties Questionnaire) were used as a standard tool (31,32). The aim of the study was to investigate the correlation between the scores of standard questionnaires and the designed questionnaire. The study was conducted with the permission of the Ethics Committee of Tehran University of Medical Sciences. In order to collect information, along with the necessary coordination with the General Directorate of Education in Qazvin Province, the consent form was completed by students and their parents.

## Results

The mean age of participants was 15.12 ± 15.3 yr. 49.90% of the participants were female and 50.10% were male. The results of each process were as follows:

### Development of questionnaire

After conclusion, the results of the qualitative study of The Iranian Adolescent Academic Stress Questionnaire (IAASQ) was designed with 75 questions in various domains of stress sources such as family conditions, interaction with teachers, educational system, peer pressure, future concerns and financial problems. Due to the similarity of the structure of the ASQ questionnaire with the objectives of the present study, the questions of the IAASQ questionnaire were adapted with the ASQ questionnaire. In case of similar questions, the questions of ASQ questionnaire were used. In addition to the similar questions, the questions included in the IAASQ questionnaire according to the participants in the interviews are as follows: 4 questions for each of the home life and the School Performance domains, 5 questions for interaction with teacher, 2 questions for each of the Peer Pressure and the future uncertainty domains were considered in the IAASQ questionnaire. In the domain of school attendance, similar questions were used. Moreover, other questions as the sources of adolescent stress about adulthood financial responsibilities and leisure time were included in IAASQ.

### Content Validity

After applying the experts’ opinion about the validity of the content of the draft version, 14 questions were deleted and 4 questions were merged with the remained, and IAASQ questions were reduced to 57 questions. Indicators of relevancy and clarity of the whole tool were obtained as 0.81 and 0.83, respectively.

### Reliability

The Cronbach’s alpha value was in the range of 0.58 to 0.85 for the domains of the questionnaire. Except the domains of interaction with teachers (0.58) and school rules (0.61), other domains were in an acceptable range, indicating that the internal consistency of the questionnaire was acceptable (33). The ICC value for the entire questionnaire was 0.80 (95% CI: 0.66–0.90) and in the domains of the questionnaire was in the range of 0.71 and 0.88, confirming the repeatability of the findings (34) (Table 1).

**Table 1:** Reliability and Factor Analysis of Iranian Adolescent Stress Questionnaire (IAASQ)

	***Factors***	***Factor loading***	***Corrected item-total correlation***	***Cronbach’s Alpha if item deleted***	***Cronbach’s Alpha***	***Intra Class Correlation (95% CI:ICC)***
	**Factor 1—Stress of Home Life (7.84% variance)**				0.81	0.88(0.80–0.94)
S1	Arguments at home	0.65	0.25	0.82		
S2	Disagreements between your parents	0.60	0.50	0.79		
S3	Disagreements between you and your parents	0.65	0.49	0.80		
S4	Lack of understanding by your parents	0.70	0.61	0.78		
S6	Little or no control over your life	0.60	0.57	0.78		
S7	Not being taken seriously by your parents	0.64	0.65	0.77		
S8	Lack of trust from adults	0.55	0.45	0.80		
S10	Parents hassling you about the way you look	0.40	0.52	0.79		
S12	parent′s care about your talents and interests	0.65	0.54	0.79		
	**Factor 2—Stress of educational system(6.41% variance)**				0.85	0.88(0.80–0.94)
S15	Having to study things you do not understand	0.41	0.45	0.72		
S18	Having to study things you are not interested in	0.51	0.45	0.72		
S20	Pressure of study	0.41	0.70	0.68		
S21	duration of attendance at any of the classes	0.62	0.49	0.72		
S24	supplementary and educational aid books	0.52	0.46	0.72		
S25	Getting up early in the morning to go to school	0.64	0.10	0.78		
S26	Compulsory school attendance	0.74	0.49	0.71		
S27	Going to school	0.74	0.50	0.71		
	**Factor 3—Stress of Future Uncertainty (6.12% variance)**				0.80	0.84(0.72–0.92)
S23	difference between studying for school exams and university entrance exam		0.46	0.59		
S47	Concern about your future	0.76	0.50	0.58		
S48	Having to make decisions about future work or education	0.80	0.39	0.61		
S49	Putting pressure on your-self to meet your future goals	0.61	0.04	0.71		
S50	worry about failure the university entrance exam	0.62	0.55	0.55		
S51	worry about the future becuse unemployed people are in society	0.51	0.40	0.61		
	**Factor 4-academic competition (5.52% variance)**				0.73	0.87(0.78–0.94)
S22	Importance of grades of exams and rankings in the class	0.49	0.55	0.76		
S36	worry feel about the behavior of your friends and peers, in the case of educational failure	0.62	0.64	0.72		
S44	Worry feel about the behavior of teachers, deputies and school principals, in the case of educational failure	0.65	0.69	0.70		
S45	threats of the principal, deputy and teachers to expel students from the school	0.58	0.36	0.82		
S46	the announcing the names of top ranks and sticking photos of them at the school board	0.54	0.64	0.72		
	**Factor 5—Stress of Teacher Interaction (5.10% variance)**				0.58	0.78(0.61–0.89)
S16	Teachers expecting too much from you	−0.49	0.70	−0.00		
S37	Not being listened to by teachers	0.61	0.11	0.80		
S38	Getting along with your teachers	0.66	0.51	0.35		
S42	principal and teachers no pay attention to your talents and interests	0.55	0.47	0.36		
	**Factor 6—Stress of school regulations (4.23% variance)**				0.61	0.71(0.50–0.86)
S39	Disagreements between you and your teachers	0.53	0.35	0.23		
S40	Teachers hassling you about the way you look	0.57	0.01	0.55		
S41	Abiding by petty rules at school	0.58	0.34	0.26		
S43	principal, deputy and teachers don′t justice to the students	0.52	0.28	0.33		
	**Factor 7—Stress of Peer Pressure (4.2% variance)**				0.80	0.88(0.80–0.94)
S28	Pressure to fit in with peers	0.52	0.75	0.56		
S29	Being hassled for not fitting in	0.57	0.71	0.58		
S30	Peers hassling you about the way you look	0.58	0.14	0.79		
S31	Being judged by your friends	0.69	0.51	0.68		
S32	Disagreements between you and your peers	0.63	0.38	0.73		
	**Factor 8—Stress of parents involved (4.08% variance)**				0.70	0.84(0.72–0.92)
S11	parent′s involvement in your studying method	0.63	0.55	0.56		
S13	parent′s blame for your low grades	0.76	0.73	0.42		
S14	parent′s compare for your grades with your friends or other family members	0.61	0.59	0.52		
S53	Not enough time for fun and activities outside of school hours	0.49	0.04	0.81		
	**Factor 9—Stress of Financial Pressure (3.73% variance)**				0.78	0.82(0.67–0.91)
S55	Not enough money to buy the things you need	0.75	0.42	0.65		
S56	Not enough money to buy the things you want	0.75	0.63	0.35		
S57	Having to take on new family responsibilities with growing older	0.57	0.41	0.66		

### Construct Validity

After designing the questionnaire, in order to construct validity of the questionnaire the exploratory factor analysis was performed. Sampling adequacy and correlation matrix was suitable for performing EFA (KMO = 0.84, χ2 = 14914.60, *P*<.001). According to the amount of eigenvalue, scree plot, expert opinion and the variance explained (48.53%), the first 10 factors were selected to explore the structure of the questionnaire (29,30). Then, for the states of 3 to 10 factors, multiple factor analysis tests were performed with extraction through principle components (PC) and principal axis factoring (PAF) methods and rotation through varimax and promax variables methods (32 factors structure). Finally, 9 factors were extracted using principal components (PC) and varimax method. The KMO index was 0.85 and (χ2 = 14519.28, *P* <.001). The range of loading factor for the extracted factors was 0.403 to 0.797. The 9 extracted factors explained 47.21% of the total variance.

According to expert opinion and the content of the questions, the naming of extractives factors were as follows: family life conditions, educational system, future concerns, educational competition, school homework, interaction with teachers, school regulations, peer pressure, parent involvement and financial problems. The results of the exploratory factor analysis of the questionnaire are shown in Table 1.

### Confirmatory factor Analysis

The results of the analysis indicated that the observed model in the studied sample was confirmed (χ2 = 2574.39, *P* <0.001). The value of RMSEA was 0.04 (95% CI = 0.02–0.06) which was at an acceptable level (0.05) and confirms the suitability of the functional structure model (29). In all the questions (except S53), the ratio of the estimated value to its standard deviation was greater than 1.96, indicating that there is a meaningful relationship between the loading factor of each observed variable on the latent variable.

### Discriminant and Convergent Validity

As expected, the overall score of the Iranian Adolescents Academic Stress Questionnaire had a positive and negative correlation with the total score of each MJSES and SDQ questionnaires, respectively (Table 2).

**Table 2:** Discriminant and Convergent Validity of Iranian Adolescents Academic Stress Questionnaire (IAASQ)

***Variable***	***Mean (Standard Deviation)***	***ASQ-I***	***SDQ***	***SE***
ASQ-I	135.82(22.26)	1		
SDQ	13.45(5.96)	.41^**^	1	
SE	64.00(7.95)	−.08^**^	.032	1

**. Correlation is significant at the 0.01 level (2-tailed).

## Discussion

The aim of present study was to development the IAASQ. According to the results of EFA, the questionnaire (IAASQ) included 9 factors and 57 questions. The characteristics of the IAASQ show that, besides the academic stressors that are common in different countries, the lives of Iranian adolescents are influenced by different stressors due to the socio-cultural structure of the country and the type of educational system. Comparison of the questions of the IAASQ and the ASQ show that there are considerable differences between academic stresses and life experiences of Iranian and Australian adolescents, so that some factors such as the responsibility for work and the existence of romantic relationships between girls and boys were not important in Iranian society, and vice versa, the factors of “educational system”, “educational competition”, “school regulations” and “parent interference” were the stressors of adolescents(9).

The first factor in the Iranian version includes the stressors that come from the family on the adolescent, which addresses the conditions of the family and the relationships of parents with their children. Like other versions of the ASQ, this factor estimates the highest share of the variance of the questionnaire (7.84), which indicates the importance of the role of families in creating academic stress (9,19,27).

The second factor, called the educational system, refers to the role of educational content, booklets and school attendance rules. Due to the long-term presence of students in school, as expected the educational system and school play an important role in adolescent academic stress. Therefore, performing stress management programs and providing mental health for them are emphasized (35).

Considering the large number of unemployed educated people in the community, worries about the future is one of the main concerns of Iranian adolescents. The third factor of the questionnaire, in addition to the similar questions in the ASQ questionnaire, pointed also the problems of university entrance exam in Iran.

One of the academic stressors of adolescents is the interaction with teachers, considered as an independent factor in this questionnaire. Due to the need to enforce laws and regulations by school officials and the sense of independence and adolescence excitement, there is a conflict in the interaction of students with teachers and school officials that can be a source of stress for them. Another issue is the importance of ratings of schools and the desire of school officials to achieve higher ranks in the education region level, which puts students at the pressure of teachers and school officials. A similar study showed that students who had good relationships with teachers had a 54% lower probability of having psychological problems than others (36).

Peer pressure is another important factor that greatly affects adolescents considered in this questionnaire as an independent factor, like other versions of ASQ (19,27). Of course, in this questionnaire, in addition to peer pressure, academic competition was identified as sources of academic stress.

In the IAASQ questionnaire, financial pressure and adulthood responsibility are together in a factor with three questions called financial problems. Therefore, the “home life”, “peer pressure”, “financial pressure”, “teacher interaction” and “future uncertainty” factors, which are among the most important domains affecting adolescent academic stress, are present in the IAASQ. However, the number of questions of the factors in the tools are not identical, which can be due to differences in the communities studied (9,19,27). By performing CFA, the IAASQ was approved in another sample of Iranian society, which shows that the tool has good structure validity. The Cronbach Alpha of different domains (0.58–0.85) and ICC (0.80(95%CI: 0.66–0.90)) in the IAASQ is at acceptable level. The Cronbach’s alpha for the Australian and Norwegian version (ASQ-N) of the ASQ was in the range of 0.62–0.92 and 0.70–0.89 respectively. Moreover, the association of this questionnaire with MJSES and SDQ confirmed its validity. The association of Australian and Norwegian versions of the ASQ had also been confirmed by self-confidence, depression, and suicide questionnaires (9,19). Since this study was conducted at the school level, there was a potential for information bias regarding academic stress. Despite the fact that many stresses affect the life of adolescents, this study mainly focused on academic stresses, especially the gender-related stresses were not evaluated.

## Conclusion

IAASQ had a comprehensive view of the different domains of adolescent academic stress and in addition to the sources of academic stress, attention has been paid to other sources of adolescent stress that could affect academic stress. IAASQ structure indicated that the physical and social environment, as well as the individual characteristics, affect health of individuals (37). Therefore, using the results of the studies of the academic stress of adolescents with Iranian tools, effective planning could be done to reduce this problem.

## Ethical considerations

Ethical issues (Including plagiarism, informed consent, misconduct, data fabrication and/or falsification, double publication and/or submission, redundancy, etc.) have been completely observed by the authors.
